# Post-translational Control of Innate Immune Signaling Pathways by Herpesviruses

**DOI:** 10.3389/fmicb.2019.02647

**Published:** 2019-11-14

**Authors:** Jessica Carriere, Youliang Rao, Qizhi Liu, Xiaoxi Lin, Jun Zhao, Pinghui Feng

**Affiliations:** Section of Infection and Immunity, Ostrow School of Dentistry, University of Southern California, Los Angeles, CA, United States

**Keywords:** herpesviruses, immune evasion, pattern recognition receptors, signaling pathways, post-translational modification

## Abstract

Herpesviruses constitute a large family of disease-causing DNA viruses. Each herpesvirus strain is capable of infecting particular organisms with a specific cell tropism. Upon infection, pattern recognition receptors (PRRs) recognize conserved viral features to trigger signaling cascades that culminate in the production of interferons and pro-inflammatory cytokines. To invoke a proper immune response while avoiding collateral tissue damage, signaling proteins involved in these cascades are tightly regulated by post-translational modifications (PTMs). Herpesviruses have developed strategies to subvert innate immune signaling pathways in order to ensure efficient viral replication and achieve persistent infection. The ability of these viruses to control the proteins involved in these signaling cascades post-translationally, either directly *via* virus-encoded enzymes or indirectly through the deregulation of cellular enzymes, has been widely reported. This ability provides herpesviruses with a powerful tool to shut off or restrict host antiviral and inflammatory responses. In this review, we highlight recent findings on the herpesvirus-mediated post-translational control along PRR-mediated signaling pathways.

## Introduction

Herpesviruses constitute a broad family of DNA viruses that cause a wide spectrum of diseases in humans, other vertebrates and non-vertebrates as well. These viruses are characterized by a common structure consisting of linear double-stranded DNA packaged in an icosahedral nucleocapsid with a size ranging from 115 to 130 nm in diameter. The nucleocapsid is surrounded by a protein layer called tegument and a lipid bilayer envelope anchored with various glycoproteins. A hallmark of all herpesviruses is the ability to establish and maintain a lifelong latent infection in the infected host. Based on the genome sequence and biological properties, the herpesviridae family is divided into three subfamilies: alpha-herpesvirinae, beta-herpesvirinae and gamma-herpesvirinae (Table 1; for review, see Davison et al., 2009). Despite the presence of a competent immune response, each herpesvirus strain has the ability to infect specific cell types within their target organisms. Therefore, they likely have evolved various strategies to subvert and exploit antiviral and inflammatory responses. Comprehension of the innate immune responses and the corresponding viral countermeasures is crucial to understanding viral pathogenesis.

**TABLE 1:** Classification of human herpesviruses.

**Subfamily**	**Taxonomic name**	**Common name**
Alpha-herpesvirinae	HHV-1	Herpes simplex virus 1 (HSV-1)
	HHV-2	Herpes simplex virus 2 (HSV-2)
	HHV-3	Varicella-zoster virus (VZV)
Beta-herpesvirinae	HHV-5	Human cytomegalovirus (HCMV)
	HHV-6	HHV-6 vairant A or B
	HHV-7	HHV-7
Gamma-herpesvirinae	HHV-4	Epstein-Barr virus (EBV)
	HHV-8	Kaposi’s sarcoma-associated herpesvirus (KSHV)

*HHV, human herpesvirus.*

During the infection, the herpesvirus faces several lines of host defense starting with the physical and mechanical mucosal/epithelial barrier (Huard et al., 1996; Rahn et al., 2017; Thier et al., 2017). At the cellular level, the virus encounters the host immune defense, i.e., the intrinsic immunity orchestrated by restriction factors suppressing/preventing the infection and the pursuit of the viral replication cycle (Tavalai and Stamminger, 2009); and the innate immunity which allows the discrimination of “non-self” components from “self” ones and triggers signaling pathways leading to pro-inflammatory and antiviral responses through the production of interferons (IFNs). Interestingly, a few years ago, Iversen et al. (2016), reported the activation of an IFN-independent innate antiviral pathway through the detection of viral O-linked glycans that leads to the production of CXCR3 chemokines which stimulate a neutrophil-dependent antiviral response.

The innate immunity process is initiated by the detection of microbial determinants or pathogen-associated molecular patterns (PAMPs) by pattern recognition receptors (PRRs) which activate various spatially localized adaptor molecules. These receptor-adaptor pairs ultimately converge to trigger inflammatory responses and/or antimicrobial gene expression *via* two crucial families of transcription factors, nuclear factor-kappa B (NF-κB) and interferon regulatory factors (IRFs) (for review, see Thaiss et al., 2016). A wide range of PRRs have been described and classified based on their subcellular location and corresponding PAMPs. The first category includes the membrane-bound receptors, such as the Toll-like receptors (TLRs) and the C-type lectin receptors (CLRs). The second group includes the cytosolic receptors, i.e., the nucleotide oligomerization domain (NOD)-like receptors (NLRs) and the retinoic acid-inducible gene-I (RIG-I)-like receptors (RLRs). Recently, intracellular DNA sensors, including cyclic GMP-AMP synthase (cGAS), interferon gamma-inducible protein 16 (IFI16), DDX41, and hnRNPA2B1 were reported to activate innate immune responses against invading DNA viruses (Sun et al., 2013; Wu et al., 2013; Almine et al., 2017; Wang et al., 2019). These PRRs reside in anatomically distinct subcellular locations to patrol for microbial infection and provoke a highly conserved signaling cascade to defeat microbial propagation.

Post-translational modifications (PTMs) are mostly enzyme-mediated, sometimes enzyme-independent or even spontaneous modifications that regulate the folding, function, subcellular localization, stability and protein-protein interactions of the target protein. In the midst of the reported PTMs, phosphorylation and ubiquitination are among the best studied. A wide range of PTMs have been described, including SUMOylation, acetylation, deamidation, methylation, ISGylation, succinylation, carbonylation, glycation, glutamylation, hydroxylation, citrullination, nitration, palmitoylation, and sulfation. The broad spectrum of PTMs and their targets enables a dynamic and tight regulation of diverse signal transduction pathways to re-establish homeostasis under stressed conditions, such as microbial infection. Innate immunity and particularly the sensing of pathogens through the PRRs is regulated by numerous PTMs to ensure efficient signal transmission and antimicrobial response. However, a growing number of studies have reported microbe-encoded enzymes that post-translationally control cellular proteins involved in innate immunity to promote the replication and/or survival of the pathogen.

Herpesviruses have acquired the ability to dampen the innate immune inflammatory and antiviral responses by regulating the proteins involved in these pathways post-translationally. In particular, herpesviruses have been shown to regulate their phosphorylation, ubiquitination, SUMOylation, acetylation, deamidation and ISGylation, which are crucial for proper signal transmission and viral eradication. In this review, we summarize the current findings on the herpesvirus-mediated direct (i.e., *via* viral-encoded enzymes) or indirect (i.e., *via* targeting cellular enzymes) post-translational control at each level of the PRR-mediated innate immune signaling pathways.

## Prr-Mediated Sensing of Herpesviruses

Each herpesvirus contains various PAMPs that can be detected by most of PRR families, including TLRs, NLRs, RLRs, CLRs, and intracellular DNA sensors (Table 2).

**TABLE 2:** PRRs involved in the sensing of herpesviruses.

	**PRR**	**Herpesvirus**	**PAMPs**	**References**
TLRs	TLR2	EBV	dUTPase	Gaudreault et al., 2007; Ariza et al., 2009
		HCMV	gB and/or gH	Compton et al., 2003; Boehme et al., 2006
		HSV-1, HSV-2	gH/gL and gB	Kurt-Jones et al., 2004; Sorensen et al., 2008; Leoni et al., 2012
		mHV68	?	Michaud et al., 2010
		VZV	?	Wang et al., 2005
	TLR3	EBV	EBV-encoded small RNA	Iwakiri et al., 2009
		HCMV	dsRNA	Nahum et al., 2012
		HSV-1	dsRNA	Zhang et al., 2007; Guo et al., 2011
		KSHV	dsRNA	West and Damania, 2008
		MCMV	dsRNA	Tabeta et al., 2004
	TLR4	HSV-2	?	Liu et al., 2014
		KSHV	?	Lagos et al., 2008
	TLR7	EBV	ssRNA	Martin et al., 2007
		HSV-1	ssRNA	Li et al., 2006
		MCMV	ssRNA	Zucchini et al., 2008
	TLR9	EBV	Genomic DNA	Lim et al., 2007; Fiola et al., 2010
		HCMV	Genomic DNA	Varani et al., 2007
		HSV-1, HSV-2	Genomic DNA	Lund et al., 2003; Krug et al., 2004; Rasmussen et al., 2009
		KSHV	Genomic DNA	West et al., 2011
		MCMV	Genomic DNA	Krug et al., 2004; Tabeta et al., 2004; Delale et al., 2005
		VZV	Genomic DNA	Yu et al., 2011
NLR and PYHIN families	IFI16^∗^	BoHV-1	Genomic DNA	Wang J. et al., 2014
		EBV	Genomic DNA	Ansari et al., 2013
		HCMV	Genomic DNA	Horan et al., 2013
		HSV-1, HSV-2	Genomic DNA	Unterholzner et al., 2010; Horan et al., 2013; Johnson et al., 2013
		KSHV	Genomic DNA	Kerur et al., 2011; Singh et al., 2013; Roy et al., 2016
	NOD1, NOD2	HCMV	?	Kapoor et al., 2014; Fan et al., 2016
	NLRP3	BoHV-1	?	Wang J. et al., 2014
		HSV-1	?	Muruve et al., 2008; Johnson et al., 2013
		MCMV	DAMP (ROS)	Zhuang et al., 2018
		MHV68	?	Sun et al., 2015
		VZV	?	Nour et al., 2011
	AIM2	HCMV	Genomic DNA	Huang et al., 2017
		MCMV	Genomic DNA	Rathinam et al., 2010
	NLRC3	HSV-1	Genomic DNA	Li et al., 2019
RLRs	RIG-I/RNA PolIII	EBV	EBV-encoded small RNA	Samanta et al., 2006, 2008; Ablasser et al., 2009
		HSV-1	dsRNA	Chiu et al., 2009; Rasmussen et al., 2009
		KSHV	Viral transcripts	Zhang Y. et al., 2018
	MDA5	HSV-1	dsRNA	Melchjorsen et al., 2010
CLRs	DC-SIGN	HCMV	gB	Halary et al., 2002
		HSV-1, HSV-2	gB and gC	de Jong et al., 2008
		KSHV	?	Rappocciolo et al., 2006
Intracellular DNA sensors	IFI16^∗^	BoHV-1	Genomic DNA	Wang J. et al., 2014
		EBV	Genomic DNA	Ansari et al., 2013
		HCMV	Genomic DNA	Horan et al., 2013
		HSV-1, HSV-2	Genomic DNA	Unterholzner et al., 2010; Horan et al., 2013; Johnson et al., 2013
		KSHV	Genomic DNA	Kerur et al., 2011; Singh et al., 2013; Roy et al., 2016
	cGAS	EBV	Genomic DNA	Wu et al., 2015
		HCMV	Genomic DNA	Paijo et al., 2016
		HSV-1	Genomic DNA	Li et al., 2013; Orzalli et al., 2015
		KSHV	Genomic DNA	Wu et al., 2015
	DAI (ZBP1)	HCMV	Genomic DNA	DeFilippis et al., 2010
		HSV-1	Genomic DNA	Takaoka et al., 2007

*The different PRRs responsible for the sensing of herpesviruses are presented, along with the ligand/PAMP detected. AIM2, absent in melanoma 2; BoHV-1, Bovine herpesvirus-1; cGAS, cyclic GMP-AMP (cGAMP) synthase; CLR, C-type lectin receptor; DAI, DNA-dependent activator of interferon-regulatory factors; DAMP, danger-associated molecular pattern; DC-SIGN, dendritic cell-specific intercellular adhesion molecule-3 (ICAM-3)-grabbing non-integrin dsRNA, double-stranded RNA; EBV, Epstein-Barr virus; HCMV, human cytomegalovirus; HSV, herpes simplex virus; IFI16, gamma interferon-inducible protein 16; KSHV, Kaposi sarcoma–associated herpesvirus; MCMV, murine cytomegalovirus; MDA5, melanoma differentiation-associated protein 5; NLR, nucleotide oligomerization domain (NOD)-like receptor; NLRC3, NOD-like receptor family CARD domain-containing 3; NLRP3, NOD-like receptor family pyrin domain-containing 3; PAMP, pathogTable 2en-associated molecular pattern; RIG-I, retinoic acid-inducible gene-I; RLR, RIG-I-like receptor; RNA Pol III, RNA polymerase III; ROS, reactive oxygen species; ssRNA, single-stranded; TLR, Toll-like receptor; VZV, varicella zoster virus; ZBP1, Z-DNA binding protein 1; ?, unindentified PAMP. ^∗^The intracellular DNA sensor IFI16 also belongs to the PYHIN family.*

### TLR Signaling Pathway

TLRs, the first discovered and best characterized PRRs, are transmembrane proteins found on the cell surface and in the endosomal membrane. TLRs constitute a family of receptors sharing sequence homology. To date, 10 TLRs in human and 13 in mouse have been described (for review, see Botos et al., 2011). Among these, TLR2, TLR3, TLR4, TLR7, and TLR9 have been reported to directly sense or indirectly participate in innate immune defense against herpesvirus infection (Table 2; Ma and He, 2014; Reuven et al., 2014). These receptors contain two key domains, a leucine-rich repeat (LRR) domain located in the extracellular or endosomal compartment, and a Toll/interleukin-1 receptor (TIR) domain in the cytoplasm. Upon the LRR-dependent binding to a ligand, TLRs transmit signal across the membrane through the TIR domain, recruiting downstream adaptor proteins such as myeloid differentiation primary response protein 88 (MyD88), MyD88 adaptor-like protein (Mal), Toll/interleukin 1 receptor domain-containing adaptor protein (TIRAP), Toll/interleukin 1 receptor domain-containing adaptor-inducing IFN-β (TRIF), and/or TRIF-related adaptor molecule (TRAM). TLR-mediated adaptor oligomerization triggers the formation and activation of two kinase complexes, i.e., TANK-binding kinase 1 (TBK1) and inhibitor of kappa B (IκB) kinase (IKK). TBK1 and IKK in turn activate the transcription factors IRFs and NF-κB *via* phosphorylation, which trigger the production of pro-inflammatory cytokines (for review, see Vidya et al., 2018). Activated IRFs and NF-κB, along with activating transcription factor-2 (ATF-2)/c-Jun, the histone acetyltransferase p300 and CREB-binding protein (CBP), are part of the IFN-β enhanceosome complex responsible for the transcriptional activation of the IFN-β gene (Falvo et al., 2000; Li et al., 2000; Lin et al., 2001; Panne et al., 2007). Dendritic cells (DCs) including plasmacytoid and conventional DCs were also shown to be critical in the production of IFNs and cytokines in response to microbial infection. Hence, many studies have reported a critical role of DCs in the control of herpesviruses such as herpes simplex virus 1 (HSV-1; Siegal et al., 1999), HSV-2 (Stout-Delgado et al., 2008), human cytomegalovirus (HCMV; Kvale et al., 2006; Schneider et al., 2008; Cederarv et al., 2009), Epstein-Barr virus (EBV; Lim et al., 2007) and murine cytomegalovirus (MCMV; Dalod et al., 2002; Andoniou et al., 2005; Puttur et al., 2016). These cells sense the presence of herpesviruses through TLR7 and/or TLR9 on endosomes to induce the production of type I IFNs and other cytokines.

### NLR Signaling Pathway and Inflammasomes

NLRs are intracellular sensors located in the cytoplasm and can be activated by either PAMPs or danger-associated molecular patterns (DAMPs). The NLR family is composed of 23 members sharing a similar structure with a central NOD domain, a C-terminal LRR domain and an N-terminal binding region which may be a caspase-recruitment domain (CARD), a pyrin domain (PYD) or a baculovirus inhibitor of apoptosis protein repeat domain (BIR) (for review, see Kersse et al., 2011). The NLRs can be divided into two subfamilies known as the “inflammasome” and the “non-inflammasome” NLRs, depending on their ability to induce the formation of multiprotein complexes called inflammasomes. Inflammasomes consist of the apoptosis-associated speck-like protein containing a CARD (ASC), pro-caspase 1 and an oligomerized member of the NLR family. Inflammasome activation ultimately leads to the proteolytic cleavage and activation of caspase-1, which processes the pro-inflammatory cytokines IL-1β and IL-18 and promotes their secretion (for review, see Lamkanfi and Dixit, 2014). Several PRRs, most of them belonging to the NLR family such as NOD-like receptor family pyrin domain-containing 3 (NLRP3), or the IFI20X/IFI16 (PYHIN) family such as absent in melanoma 2 (AIM2) and IFI16, can trigger the assembly of inflammasomes. During herpesvirus infection, the assembly and activation of NLRP3, AIM2 and IFI16 inflammasomes has been reported upon the detection of various viral PAMPs or DAMPs (Table 2). Moreover, a recent study showed that dsDNA from HSV-1 binds to NOD-like receptor family CARD domain-containing 3 (NLRC3), leading to the detachment of NLRC3 from stimulator of interferon genes (STING) that is then available to activate the IFN pathway (Li et al., 2019). Among the “non-inflammasome” NLRs, NOD1, and NOD2 are the most studied and have been shown to induce the formation of another multiprotein complex, the NODosome, which in turn activates IRF, NF-κB, and MAPK in response to viral infection (for review, see Ting et al., 2010). To date, only HCMV has been shown to be detected by NOD1 and NOD2 though the ligand has not been identified yet (Kapoor et al., 2014; Fan et al., 2016).

### RLR Signaling Pathway

RLRs are intracellular sensors responsible for the detection of viral dsRNA in the cytoplasm (for review, see Reikine et al., 2014). The RLR family is composed of three homologous members, RIG-I or DExD/H-Box helicase 58 (DDX58), melanoma differentiation-associated protein 5 (MDA5) or IFN-induced with helicase domain 1 (IFIH1) and laboratory of genetics and physiology 2 (LGP2) or DExH-Box helicase 58 (DHX58). These proteins share a similar structure with a central DExH/D-box RNA helicase domain responsible for binding dsRNA and a C-terminal domain (CTD). However, unlike LGP2, RIG-I, and MDA5 possess two additional N-terminal CARD domains that can dimerize with the CARD domain of RIG-I, MDA5 or mitochondrial antiviral signaling protein (MAVS). Despite their sequence and structural similarities, these proteins recognize distinct features of viral dsRNA. RIG-I detects short dsRNA with a 5′-tri- or di-phosphate moiety, while MDA5 recognizes long dsRNA with no end-specificity (for review, see Bruns and Horvath, 2014). Interestingly, some herpesviruses such as HSV-1, EBV and Kaposi sarcoma-associated herpesvirus (KSHV), produce dsRNA that can be detected by RIG-I, as the RNA-Pol III converts viral DNA into RNA containing 5′ tri-phosphate moiety (Samanta et al., 2006, 2008; Ablasser et al., 2009; Chiu et al., 2009; Rasmussen et al., 2009; Melchjorsen et al., 2010; Zhang Y. et al., 2018). Recently, several studies showed that RIG-I is able to directly recognize viral or host dsRNA species which are not of DNA origin (Samanta et al., 2006; Cheng et al., 2007; Rasmussen et al., 2007; Chiu et al., 2009; West et al., 2014; Liu et al., 2016; Zhang Y. et al., 2018; Zhao et al., 2018; Lee et al., 2019). In resting cells, RIG-I is maintained in an autoinhibited conformation characterized by the intramolecular interaction between the CARD domain and the helicase 2 insertion (Hel2i) domain (Kowalinski et al., 2011; Luo et al., 2011), thereby exposing the CTD to patrol the cytoplasm for microbial dsRNA (Jiang et al., 2011). The binding of dsRNA by CTD triggers an overall conformation change of RIG-I, which coils around the dsRNA helix to form a “O” ring-like structure. In doing so, RIG-I exposes the CARD domain, so it is free to interact with the CARD domain of MAVS, promoting the transcription and production of IFNs and inflammatory cytokines (Kawai et al., 2005; Seth et al., 2005; Xu et al., 2005; Cui et al., 2008; Takahasi et al., 2008). Interestingly, dsRNA binding to MDA5 induces CTD rotation, which triggers the formation of an oligomeric MDA5 filament on the dsRNA. Filament formation of MDA5 releases its CARD domains, which heterodimerize with the CARD of MAVS (Peisley et al., 2011; Berke and Modis, 2012; Reikine et al., 2014). Lacking CARD domains, LGP2 cannot induce downstream innate immune signaling. Perplexingly, LGP2 has been reported as being able to act either as a negative regulator of the recognition of viral RNA by RIG-I or as a positive regulator of RIG-I- and MDA5-mediated viral dsRNA recognition and antiviral signaling (Yoneyama et al., 2005; Satoh et al., 2010; Childs et al., 2013; Bruns and Horvath, 2015). The opposing effect of LGP2 on dsRNA-mediated innate immune signaling may be context-dependent. Nevertheless, these results suggest that LGP2 is a regulatory homolog of RIG-I and MDA5.

### CLRs Signaling Pathway

CLRs are selectively expressed on the surface of immune cells such as Langerhans cells, monocytes, macrophages and DCs. These proteins specifically recognize carbohydrate moieties to sense pathogens. Binding to carbohydrates triggers the internalization and typically the degradation of CLRs *via* the lysosomal pathway (for review, see Bermejo-Jambrina et al., 2018). Given their presence on immune cells, these CLRs are able to trigger both innate and adaptive (*via* the presentation of microbial antigens) immune responses. Some CLRs such as immunoreceptor tyrosine-based activation motifs (ITAMs) in Dectin-2, DC immune-activating receptor (DCAR) and myeloid DAP12-associated lectin-1 (MDL-1) and immunoreceptor tyrosine-based inhibition motifs (ITIMs) in DC immunoreceptor (DCIR), contain distinct signaling motifs. Other CLRs such as DC-specific intercellular adhesion molecule-3 (ICAM-3)-grabbing non-integrin (DC-SIGN), mannose receptor (MR) and lymphocyte antigen 75 (LY75), contain no signaling motifs in their cytoplasmic tails at all. These motifs provide a structural and physical platform to enable the crosstalk with the immune pathways triggered by other PRRs. Very few studies have implicated roles of CLRs during herpesvirus infection. DC-SIGN and the related receptor DC-SIGNR have been reported to be activated by HSV-1, HSV-2, KSHV, and HCMV (Table 2; Halary et al., 2002; Rappocciolo et al., 2006; de Jong et al., 2008). To date, the post-translational control of CLR-mediated recognition of herpesviruses remains unknown.

### Intracellular DNA Sensors

Recently, several intracellular DNA sensors have been identified, including cGAS, IFI16, DNA-dependent activator of IFN-regulatory factors (DAI), AIM2, DEAD-box helicase 41 (DDX41), Z-DNA binding protein 1 (ZBP1), and RNA polymerase III (Takaoka et al., 2007; Chiu et al., 2009; Hornung et al., 2009; Unterholzner et al., 2010; Parvatiyar et al., 2012). Further studies have reported a pivotal role for cGAS in sensing cytosolic DNA. Upon detection of cytoplasmic DNA, cGAS undergoes dimerization and a structural rearrangement centered on the catalytic core that synthesizes cGAMP, a second messenger that in turn triggers a conformational change and activation of STING (Li et al., 2013; Sun et al., 2013; Zhang X. et al., 2013). Activated STING recruits TBK1 and IRF3 to facilitate the phosphorylation of IRF3 by TBK1 and the subsequent production of IFN-β (Ishikawa and Barber, 2008; Unterholzner et al., 2010; Abe et al., 2013; Unterholzner, 2013; Wu et al., 2013; Almine et al., 2017). Compared to other nucleic acid sensors, such as RIG-I for dsRNA, the DNA-binding affinity of cGAS appears to be low; the potent enzymatic activity in cGAMP synthesis may compensate for this (Li et al., 2013). Alternatively, a growing amount of studies highlighted the importance of the post-translational control of cGAS in the establishment of a proper and regulated antiviral response. Hence, cGAS stability, enzymatic activity and binding to dsDNA is tightly regulated by phosphorylation (Seo et al., 2015), ubiquitination (Bhoj and Chen, 2009; Chen et al., 2016; Wang et al., 2017), SUMOylation (Hu et al., 2016; Cui et al., 2017) and glutamylation (Xia et al., 2016). Unlike the other DNA sensors, IFI16 is primarily located in the nuclei of resting cells. Upon HSV-1 infection, IFI16 interacts with the viral genome in the nucleus and is acetylated by the histone acetyltransferase p300, thus triggering its translocation into the cytoplasm where it binds to STING to induce IFN production (Unterholzner et al., 2010; Ansari et al., 2015). IFI16 can also activate a caspase 1-dependent inflammasome that processes and promotes the production of IL-1β and IL-18 (for review, see Dempsey and Bowie, 2015). cGAS, IFI16 and DAI have been shown to detect the genomic dsDNA of herpesviruses and in particular EBV, HCMV, HSV-1, HSV-2, KSHV, and bovine herpesvirus-1 (BoHV-1; Table 2; Takaoka et al., 2007; DeFilippis et al., 2010; Unterholzner et al., 2010; Kerur et al., 2011; Ansari et al., 2013; Horan et al., 2013; Johnson et al., 2013; Li et al., 2013; Singh et al., 2013; Wang J. et al., 2014; Orzalli et al., 2015; Wu et al., 2015; Paijo et al., 2016; Roy et al., 2016).

Interestingly, several studies revealed a dynamic interaction between DNA- and RNA- sensing pathways. Hence, upon HSV-1 infection and recognition of the viral 5′ tri-phosphorylated RNA by RIG-I, RIG-I upregulates STING through the NF-κB and JAK/STAT cascades (Liu et al., 2016; Zevini et al., 2017). This study also demonstrated that a proper antiviral response to HSV-1 infection *in vivo* through RIG-I signaling requires STING, revealing STING as a central molecule of RNA- and DNA-sensing pathways. However, Wu et al. (2017), demonstrated that the transfection of dsDNA into human diploid cells leads to the proteasome-mediated degradation of STING associated with an upregulation of RIG-I and IL-6 expression. In this study, the authors also showed that RIG-I and IL-6 are responsible for STING degradation, providing a negative feedback mechanism to limit the activation of STING-mediated innate immune signaling (Wu et al., 2017). This mechanism, however, seems to be limited to human diploid cells as it was not observed in HEK293 cells, suggesting that it might be a cell type-specific regulatory mechanism.

## Post-Translational Control of PRR-Mediated Signaling Pathways by Herpesviruses

Herpesviruses prevent pro-inflammatory and antiviral responses by regulating the phosphorylation, ubiquitination, SUMOylation, acetylation, deamidation and ISGylation of proteins involved in PRR-mediated signaling pathways. Phosphorylation, the most common PTM, is induced by kinases and reversed by phosphatases. Phosphorylation is critical for the regulation of numerous biological processes including cell cycle, cell growth, apoptosis, metabolism and signal transduction (for review, see Ardito et al., 2017). Phosphorylation is required for a proper signal transduction along innate immune signaling pathways. Therefore, herpesviruses have evolved to regulate the phosphorylation of critical signaling proteins either by expressing kinases inducing aberrant phosphorylation or by inhibiting cellular kinases (Table 3).

**TABLE 3:** Post-translational control of PRR-mediated signaling pathways by herpesviruses.

	**Target protein**	**Herpesvirus**	**Viral regulators**	**Regulation**	**Signaling function**	**References**
PRRs	RIG-I	EBV	BPLF1	Reduced ubiquitination	EBV BPLF1 interacts with TRIM25 and promotes its autoubiquitination and inactivation, thereby blocking RIG-I activation	Gupta et al., 2018
		HSV-1	UL37	Deamidation	The HSV-1 tegument protein UL37 deamidates RIG-I, blocking its activation	He et al., 2015; Zhao J. et al., 2016
		KSHV	ORF64	Reduced ubiquitination	KSHV deubiquitinase ORF64 inhibits RIG-I activation by reducing its ubquitination	Inn et al., 2011
		KSHV	k-vGAT	Deamidation	KSHV-encoded vGAT induces the deamidation and activation of RIG-I	He et al., 2015
		MHV68	vGAT	Deamidation	MHV68 vGAT recruits the cellular GAT PFAS to deamidate and activate RIG-I, which is necessary for RelA degradation and to avoid the production of inflammatory cytokines	He et al., 2015
	DC-SIGN, DC-SIGNR	KSHV	K3, K5	Ubiquitination/proteasome-dependent degradation	KSHV ubiquitin ligases K3 and K5 target DC-SIGN and DC-SIGNR for ubiquitination and proteasome-dependent degradation	Lang et al., 2013
	IFI16	HSV-1	ICP0	Ubiquitination/proteasome-dependent degradation	HSV-1 E3 ubiquitin ligase ICP0 promotes the ubiquitin/proteasome-dependent degradation of IFI16	Orzalli et al., 2012
	cGAS	HSV-1	UL37	Deamidation	The HSV-1 tegument protein UL37 deamidates cGAS to block cGAS-mediated signaling	Zhang J. et al., 2018
Adaptor proteins	MyD88/Mal/TIRAP	HSV-1	ICP0	Ubiquitination/proteasome-dependent degradation	HSV-1 E3 ligase ICP0 ubiquitinates MyD88 and Mal (TIRAP), leading to their proteasome-mediated degradation	van Lint et al., 2010
		KSHV	RTA	Ubiquitination/proteasome-dependent degradation	KSHV protein RTA induces the uniquitin/proteasome-dependent degradation of MyD88	Zhao et al., 2015
	TRAF3	HSV-1	UL36USP	Reduced ubiquitination	HSV-1 UL36USP deubiquinates TRAF3 ubiquitination, which is no longer recruited to TBK1	Wang et al., 2013a
	TRAF6	EBV	LMP1	Reduced ubiquitination	EBV LMP1 deubiquitinates TRAF6, blocking its activation and NF-κB signaling	Saito et al., 2013
		HSV-1	US3	Reduced ubiquitination	HSV-1 US3 reduces TRAF6 ubiquitination required for its activation and for TLR2-mediated signaling	Sen et al., 2013
		HSV-1	ICP0	Reduced ubiquitination	HSV-1 ICP0 triggers the translocation of the E3 ubiquitin ligase USP7 from the nucleus to the cytoplasm which will deubiquitinate TRAF6, inhibiting the TLR-mediated NF-κB signaling	Daubeuf et al., 2009
	STING	HSV-1	γ_1_34.5	Reduced phosphorylation	HSV-1 γ134.5 protein inhibited STING phosphorylation and activation, likely via a targeted phosphatase activity	Pan et al., 2018
		KSHV	vIRF1	Reduced phosphorylation	KSHV vIRF1 directly interacts with STING, blocking its interaction with TBK1, inhibiting TBK1 phosphorylation and activation of STING and the subsequent production of IFN-β	Ma et al., 2015
		HCMV	UL48	Reduced ubiquitination	HCMV UL48 deubiquitinase enzyme reduces K63-linked ubiquitination of STING, thus blocking STING activation	Kumari et al., 2017
		MHV68	ORF64	Reduced ubiquitination	MHV68 deubiquitinase ORF64 prevents the activation of the STING signaling pathway	Sun et al., 2015
IRFs	IRF3	EBV	BGLF4	Phosphorylation	EBV BGLF4 phosphorylates IRF3 at Ser123, Ser173, and Thr180, inhibiting its recruitment to ISREs	Wang et al., 2009
		HSV-1	US11	Reduced phosphorylation	HSV-1 US11 directly interacts with RIG-I and MDA5, impeding IRF3 activation by reducing its phosphorylation and dimerization	Xing et al., 2012
		HSV-1	US3	Hyperphosphorylation	HSV-1 US3 interacts with and hyperphosphorylates IRF3 at Ser175 to prevent its activation	Wang et al., 2013b
		HSV-1	ICP34.5	Reduced phosphorylation	HSV-1 ICP34.5 directly interacts with TBK1, blocking the phosphorylation of IRF3	Verpooten et al., 2009; Ma et al., 2012
		HSV-1	VP24	Reduced phosphorylation	HSV-1 VP24 inhibits the interferon stimulatory DNA-mediated phosphorylation and dimerization of IRF3	Zhang et al., 2016
		HSV-1	ICP27	Reduced phosphorylation	HSV-1 ICP27 interacts with STING, blocking TBK1-mediated phosphorylation of IRF3	Christensen et al., 2016
		KSHV	miR-K12-11	Reduced phosphorylation	KSHV-encoded miR-K12-11 inhibits IRF3 phosphorylation by targeting IKKε	Liang et al., 2011
		HSV-1	ICP0	Ubiquitination/proteasome-dependent degradation	HSV-1 E3 ubiquitin ligase ICP0 promotes the ubiquitin/proteasome-dependent degradation of IRF3	Melroe et al., 2004
		VZV	ORF61	Ubiquitination/proteasome-dependent degradation	VZV ORF61 directly interacts with activated IRF3, and IRF3 is ubiquitinated and downregulated in the presence of ORF61	Zhu et al., 2011
	IRF7	EBV	LMP1	SUMOylation	EBV LMP1 inhibits IRF7 by inducing its SUMOylation	Bentz et al., 2012
		KSHV	RTA	Ubiquitination/proteasome-dependent degradation	KSHV RTA promotes the ubiquitin/proteasome-dependent degradation of IRF7	Yu et al., 2005
		EBV	LMP1	Ubiquitination/proteasome-dependent degradation	EBV LMP1 promotes the TRAF6-mediated ubiquitination/proteasome-dependent degradation of IRF7	Ning et al., 2008
	IRF9	VZV	ORF63	Ubiquitination/proteasome-dependent degradation	VZV ORF63 reduces the levels of IRF9 in a proteasome degradation-dependent pathway	Verweij et al., 2015
	IRF1, IRF2, IRF7	KSHV	K-bZIP	SUMOylation	KSHV encodes a SUMO E3 ligase named K-bZIP potentially targeting the transcription factors IRF1, 2, and 7	Chang et al., 2010, 2013
	IRFs	KSHV	vIRF1	Reduced acetylation	vIRF1 directly interacts with p300 and reduces its histone acetyltransferase activity, affecting the formation of the CBP/p300 enhanceosome complex responsible for the cellular IRF transcriptional activity	Li et al., 2000; Lin et al., 2001
NF-κB	IKKβ	HCMV	UL26	Reduced phosphorylation	HCMV UL26 blocks the phosphorylation and activation of IKKβ, which is the key step for IκB phosphorylation and NF-κB activation	Mathers et al., 2014
	IκB	VZV	ORF61	Reduced ubiquitination	VZV ORF61 blocks IκB ubiquitination, inhibiting NF-κB signaling	Whitmer et al., 2015
		HSV-1	UL36USP	Reduced ubiquitination	HSV-1 UL36USP deubiquitinates IκBα, preventing it from degradation and blocking NF-κB in an inactivated form	Ye et al., 2017
	p65/RelA	HSV-1	US3	Hyperphosphorylation	HSV-1 US3 hyperphosphorylates p65 at Ser75, blocking its nuclear translocation	Wang K. et al., 2014
		MHV68	n.d.	Phosphorylation	MHV68 hijacks RIG-I and MAVS to activate IKKβ, inducing RelA phosphorylation at Ser468 and subsequent RelA degradation by the proteasomal pathway	Dong and Feng, 2011
		MHV68	ORF73	Ubiquitination/proteasome-dependent degradation	MuHV-4 ORF73 interacts *via* its SOCS box motif with ElonginC and Cullin5 to mediate p65/RelA ubiquitination and degradation	Rodrigues et al., 2009
	p50	HSV-1	ICP0	Ubiquitination/proteasome-dependent degradation	HSV-1 ICP0 interacts directly with p50 and p65/RelA, blocks the nuclear translocation of p65/RelA and induces the ubiquitination and proteasome-dependent degradation of p50	Zhang J. et al., 2013
JAK/STAT	TYK2	EBV	LMP1	Reduced phosphorylation	EBV LMP-1 directly interacts with TYK2, blocking its phosphorylation/activation and subsequent IFNα signaling	Geiger and Martin, 2006
	JAK	HSV-1	VR3, UL13, UL41	Reduced phosphorylation	HSV-1 VR3, UL13, and UL41 induce the expression of SOCS1 and SOCS3 protein to inhibit JAK phosphorylation	Chee and Roizman, 2004; Yokota et al., 2004; Sato et al., 2017
	STAT1	EBV	BZLF1	Reduced phosphorylation	EBV BZLF1 inhibits IFN-gamma-induced STAT1 tyrosine phosphorylation	Morrison et al., 2001
		HSV-1	ICP27	Reduced phosphorylation	HSV-1 ICP27 downregulates STAT1 phosphorylation and its accumulation in the nucleus	Johnson et al., 2008
	STAT2	MCMV	pMP27	Ubiquitination/proteasome-dependent degradation	pM27 induces STAT2 ubiquitination and degradation by the proteasome, likely through its interaction with DDB1	Trilling et al., 2011
		VZV	ORF63	Reduced phosphorylation	VZV ORF63 interferes with JAK-STAT signaling by reducing the IFN-induced STAT2 phosphorylation	Verweij et al., 2015
ISGylation	UL26	HCMV	IE1, UL26	Reduced ISGylation	In response to infection, HCMV pUL26 is ISGylated, destabilizing the protein and inhibiting its ability to restrict the NF-κB response. HCMV IE1 and pUL26 can suppress infection-induced ISGylation	Kim et al., 2016
	pUL50	HCMV	UBE1L	Reduced ISGylation	HCMV IE1 and UL26 are able to suppress the infection-induced ISGylation	Lee et al., 2018
	Global ISGylation	KSHV	vIRF1	Reduced ISGylation	vIRF1 interacts with the ISG15 E3 ligase HERC5, leading to a global decreased ISGylation of proteins in infected cells	Jacobs et al., 2015

*The target protein, the viral protein responsible for the regulation, the PTM involved, and the signaling function/consequence of the regulation are presented. CBP, CREB-binding protein; DC-SIGN, dendritic cell-specific intercellular adhesion molecule-3 (ICAM-3)-grabbing non-integrin; DC-SIGNR, DC-SIGN receptor; DDB1, DNA binding protein 1; EBV, Epstein-Barr virus; GAT, glutamine amidotransferase; vGAT, viral GAT; HSV, herpes simplex virus; HERC5, HECT and RLD domain containing E3 ubiquitin protein ligase 5; HCMV, human cytomagalovirus; IFI16, gamma interferon-inducible protein 16; IFN, interferon; IκB, inhibitor of kappa B; IKKe, IκB kinase; IRF, interferon regulatory factor; ISG, interferon-stimulated gene; ISREs, interferon-sensitive response elements; JAK, janus kinase; K-bZIP, KSHV basic region-leucine zipper; KSHV, Kaposi sarcoma–associated herpesvirus; LMP1, latent membrane protein 1; Mal, MyD88 adaptor-like protein; MDA5, melanoma differentiation-associated protein 5; miR, microRNA; MyD88, myeloid differentiation primary response protein 88, NF-κB, nuclear factor-kappa B; ORF, open reading frame; PFAS, phosphoribosylformylglycinamidine synthetase; RIG-I, retinoic acid-inducible gene-I; RTA, replication and transcription activator; SOCS, suppressor of cytokine signaling; STAT, signal transducer and activator of transcription; STING, stimulator of IFN genes; TBK1, TANK-binding kinase 1; TIRAP, Toll/interleukin 1 receptor domain-containing adaptor protein; TRAF, tumor necrosis factor (TNF) receptor-associated factor; TRIM, tripartite motif; TYK2, non-receptor tyrosine kinase 2; UL36USP, UL36 ubiquitin-specific protease; USP7, ubiquitin specific peptidase 7; VZV, varicella zoster virus.*

Ubiquitination corresponds to the attachment of ubiquitin moiety to lysine residues of a target protein and requires the sequential action of three enzymes: an E1 ubiquitin-activating enzyme, an E2 ubiquitin-conjugating enzyme, and an E3 ubiquitin ligase. The ubiquitination process starts with the attachment of a monoubiquitin to the target residue and can remain as monoubiquitination or be further extended with the attachment of additional ubiquitin molecules, forming elongated polyubiquitin chains. The conventional linkage of ubiquitin occurs on lysine residues of the target protein although unconventional linkages to cysteine residues have also been identified (for review, see McDowell and Philpott, 2013). Both monoubiquitination and polyubiquitination are essential in the regulation of biological processes as they define the fate of a target protein depending on the lysine residue conjugated to the carboxyl terminal di-glycine of the ubiquitin. Hence, for instance, mono-ubiquitination has been associated with the regulation of protein trafficking and subcellular localization, or chromatin regulation while polyubiquitination has been associated with protein clearance though the proteasomal or autophagic pathways and with protein signaling (for review, see Husnjak and Dikic, 2012). The fate of a target protein is further regulated by ubiquitin chain linkage or topology. Ubiquitin itself contains seven lysine residues (K6, K11, K27, K29, K33, K48, K63, and M1) that can be ubiquitinated and the role of K48 and K63, to a less extent K27, is well established. Specifically, the K48-linked polyubiquitin chain generally targets proteins for proteasomal degradation, while a polyubiquitin chain of the K63-linkage can regulate signal transduction or protein trafficking (for review, see Pickart and Eddins, 2004). Given the critical role of this PTM in the regulation of innate immune signaling pathways, it is not surprising that herpesviruses exploit ubiquitination to dampen the cellular antiviral response. Notably, herpesviruses express proteins that possess the intrinsic activities of either E3 ubiquitin ligases or deubiquitinases (Table 3).

SUMOylation has emerged as a critical player in the regulation of signaling pathways. SUMOylation occurs after the binding of a protein containing a SUMO-interacting motif (SIM). Similar to ubiquitination, SUMOylation is a cascade of reactions catalyzed by three distinct enzymes, a SUMO E1 activating enzyme, a SUMO E2 conjugating enzyme, and a SUMO E3 ligase which links a SUMO moiety to a lysine residue in the target protein. SUMOylation has diverse consequences on the target protein and plays an important role in regulating protein localization, trafficking and signal transduction (for review, see Wilkinson and Henley, 2010). Studies have reported strategies evolved by herpesviruses to dampen SUMOylation by host SUMO E3 ligases or to directly SUMOylate proteins involved in antiviral signaling (Table 3).

Acetylation is one of the major PTMs and consists of the attachment of an acetyl group from the donor acetyl-coenzyme A (Acetyl-CoA) to the target protein. This reaction is catalyzed by acetyltransferases and can either occur at the N-terminus of the target protein or at the α-amino group of lysine residues (for review, see Drazic et al., 2016). Along with the other PTMs, acetylation allows the tight regulation of numerous biological processes by regulating the function and localization of the target proteins including those involved in innate immune signaling pathways. Hence, acetylation is also controlled by herpesviruses to restrict antiviral responses (Table 3). Notably, upon infection by KSHV, EBV and HSV-1, the innate DNA sensor IFI16 interacts with the histone acetyltransferase p300 and CREB-binding protein (CBP) in the nucleus, which leads to the acetylation of IFI16. This acetylation triggers the translocation of IFI16 to the cytoplasm, where it then interacts with ASC, resulting in inflammasome assembly and increased interaction with STING. Ultimately, these signaling events activate IFN-β induction (Wathelet et al., 1998; Ansari et al., 2015; Dutta et al., 2015). Acetylation often targets lysine residues for modification like ubiquitination and SUMOylation. These PTMs may compete and target the same lysine residue to dictate the fate of the target protein. Acetylation and Acetyl-CoA provide a point of crosstalk with key cellular metabolic pathways since Acetyl-CoA intersects with glycolysis, tricarboxylic acid cycle (TCA) and lipid synthesis. A growing amount of studies demonstrated a metabolic reprogramming upon herpesvirus infection and on glycolysis, TCA cycle and lipid synthesis in particular (for review, see Thaker et al., 2019), giving insight on the critical role that Acetyl-CoA and acetylation might play in herpesvirus pathogenesis.

### Post-translational Control of PRRs

#### RIG-I

The mechanism of RIG-I activation requires K63-ubiquitination by E3 ubiquitin ligases including tripartite motif 25 (TRIM25; Gack et al., 2007) and RIPLET (Cadena et al., 2019). To fully activate RIG-I, both covalent conjugation of a polyubiquitin chain to RIG-I and free polyubiquitin chains are required to induce RIG-I oligomerization (Zeng et al., 2010; Peisley et al., 2014). RIG-I ubiquitination is a critical checkpoint to regulate its dsRNA engagement and subsequent activation. Therefore, herpesviruses demonstrate the ability to counteract this activation mechanism. For instance, KSHV open reading frame (ORF) 64 is able to disassemble these polyubiquitin chains through its deubiquitinase activity to suppress RIG-I activation and the subsequent antiviral signaling, leading to an increased lytic replication of the virus (Inn et al., 2011). Recently, Gupta et al. (2018), reported that BPLF1, encoded by the EBV genome, interacts with TRIM25 to promote its auto-ubiquitination and inactivation, thereby blocking RIG-I activation and the IFN induction. This study also revealed that the effect of BPLF1 is conserved in homolog proteins encoded by other herpesvirus families such as HSV, HCMV, and KSHV. Controlling RIG-I deamidation enabled herpesviruses to evade the immune system as well. Indeed, murid gamma-herpesvirus 4 (MuHV-4) also known as murine gamma herpesvirus 68 (MHV68), a murine model gamma-herpesvirus closely related to human KSHV and EBV, encodes homologs of cellular glutamine amidotransferases (GATs), dubbed viral GATs or vGATs. vGAT encoded by the ORF75c open reading frame lacks key active sites required for enzyme catalysis and is a pseudo-enzyme. Despite missing intrinsic enzymatic activity, vGAT can recruit cellular phosphoribosyl-formylglycinamidine synthetase (PFAS) to deamidate and activate RIG-I (He et al., 2015). While KSHV and EBV encode one vGAT, herpesvirus saimiri (HVS; a strain infecting primates) and MHV68 encode two and three vGAT homologs, respectively. Whether these additional vGAT homologs possess a similar function remains unknown. HSV-1 encodes no homolog of vGAT, however, the infection induces a shift of RIG-I toward the positive side on a two-dimensional gel electrophoresis which is an indication of deamidation. A functional screen identified that the tegument protein UL37 demonstrates intrinsic enzymatic activity to deamidate RIG-I and that this deamidation abolishes the ability of RIG-I to bind and sense dsRNA (Zhao J. et al., 2016). Retrospectively, it appears that UL37 of alpha-herpesviruses shares a similar repertoire of function with vGAT proteins of gamma-herpesviruses, despite the lack of sequence homology (Gaspar et al., 2008; Liu et al., 2008; Pitts et al., 2014; He et al., 2015). Whether beta-herpesviruses express viral proteins with similar properties is unclear. These observations reveal that herpesviruses utilize protein phosphorylation and deamidation to evade host innate immune activation upon recognition of viral RNA by RIG-I.

#### DC-SIGN and DC-SIGNR

The post-translational regulation of CLRs by herpesviruses is not well understood. However, a study revealed that the KSHV-encoded ubiquitin ligases K3 and K5 are able to target the receptors DC-SIGN and DC-SIGNR for ubiquitination and subsequent proteasome-dependent degradation (Lang et al., 2013) implying the defensive role of these receptors against KSHV infection.

#### cGAS and IFI16

cGAS and IFI16 are important in host innate immune defense and are targeted by herpesviruses to shut off the inflammatory and antiviral responses. For instance, HSV-1 infection is sensed in the nucleus by IFI16 upon release of viral DNA, which presumably triggers IFI16 acetylation and subsequent nuclear export. Cytoplasmic IFI16 then induces IFN production or inflammasome activation (Unterholzner et al., 2010; Horan et al., 2013; Johnson et al., 2013). Interestingly, IFI16 was also reported to act in the nucleus to directly suppress viral gene expression against KSHV and HCMV (Kerur et al., 2011; Singh et al., 2013; Roy et al., 2016). An unanswered question is how IFI16 distinguishes cellular from viral genomic DNA in the nucleus. Additionally, the dichotomy of IFI16 action in antiviral defense at two distinct subcellular locations against herpesvirus infection requires further investigation.

HSV-1 has evolved to regulate the ubiquitination of IFI16 and the deamidation of cGAS. Hence, the HSV-1 E3 ubiquitin ligase ICP0 promotes the ubiquitination and proteasome-dependent degradation of IFI16, thus inhibiting viral DNA detection and the consequent IRF3-mediated signaling (Orzalli et al., 2012). However, Cuchet-Lourenco et al. (2013), later demonstrated that HSV-1 ICP0 is not sufficient to destabilize IFI16 and that IFI16 seems to be required for the recruitment of the restriction factor promyelocytic leukemia protein (PML) to decrease the viral replication, suggesting the existence of an IFN-independent antiviral activity of IFI16. Interestingly, HSV-1 UL37 was recently shown to deamidate human and mouse cGAS, but not cGAS from non-human primates, to antagonize cGAS-mediated immune activation (Zhang J. et al., 2018). Deamidated cGAS, although able to interact with dsDNA and dimerize, fails to synthesize cGAMP and induce downstream signaling, allowing an increased replication of the virus. Thus, deamidation of RIG-I and cGAS conveyed by HSV-1 UL37 inactivates host innate immune activation by dsRNA and dsDNA, respectively. Importantly, a recombinant HSV-1 carrying an active site mutation within UL37 is highly attenuated in its viral replication and pathogenesis in mice. The loss of cGAS and STING restored the replication and pathogenesis of the deamidase-deficient HSV-1 in mice, demonstrating a specific role of UL37-mediated evasion from the cGAS-STING signaling pathway *in vivo*. The role of UL37-mediated evasion of RIG-I by HSV-1 remains to be determined. These studies revealed immune evasion mechanisms *via* regulating the ubiquitination and deamidation of IFI16 and cGAS respectively to restrict the recognition and subsequent antiviral signaling by these DNA sensors in the context of HSV-1 infection. However, the use of similar mechanisms by other herpesviruses is yet to be determined. Moreover, whether herpesviruses exploit or modulate cGAS-regulating PTMs i.e., phosphorylation, ubiquitination, SUMOylation, and glutamylation would be an interesting study that paves the way for identifying new immune evasion strategies of herpesviruses.

### Post-translational Control of PRR Adaptors

#### MyD88/Mal/TIRAP

Mal is a bridging adapter molecule responsible for the specific recruitment of MyD88 to the TLR2 and TLR4 complexes (Yamamoto et al., 2002; Fitzgerald et al., 2003; Kagan and Medzhitov, 2006). By targeting these adaptor proteins for degradation by the proteasomal pathway, HSV-1 and KSHV were shown to be able to restrict TLR-mediated antiviral signaling. HSV-1 ICP0 inhibits TLR2-mediated NF-κB activation by inducing the ubiquitination and subsequently the proteasomal-mediated degradation of MyD88 and Mal/TIRAP (Hagglund and Roizman, 2004; van Lint et al., 2010). KSHV can also induce the ubiquitin/proteasome-mediated degradation of MyD88 *via* its replication and transcription activator (RTA) protein and thereby inhibit TLR4 signaling (Zhao et al., 2015). Both ICP0 and RTA are expressed immediately after viral entry and belong to the immediate-early category of viral genes, consistent with the putative evasion from host defense. Unfortunately, their effect on viral lytic replication was not assessed under those conditions and remains an open question. A related study, although independent of the post-translational control of MyD88, showed that MyD88 is required for the efficient establishment of a latent MHV68 infection in B cells in a mouse model, suggesting that herpesviruses may evolve to usurp this molecule for persistent infection (Forrest and Speck, 2008). These findings highlight the function of viral proteins in the regulation of TLR signaling *via* targeting the adaptor molecules MyD88 and Mal/TIRAP.

#### TRAFs

Tumor necrosis factor (TNF) receptor-associated factor (TRAF) is a family of proteins that act as adaptor molecules in various signaling pathways. TRAF3 and TRAF6 are components of the TIR signaling complexes and are activated by conjugation of polyubiquitin chains upon TLR activation. TRAFs are critical for the induction of the type I IFN response and are recruited to the adaptors TRIF, interleukin 1 receptor-associated kinase 1 (IRAK1), and downstream of TBK1 and IKKε (for review, see Xie, 2013). Several studies have demonstrated a critical role of TRAF3 in TLR-dependent and -independent antiviral responses (Hacker et al., 2006; Oganesyan et al., 2006). As central components of TIR signaling complexes, TRAF proteins are often targeted by herpesviruses to derail the host immune defense, thus enabling their lifelong persistence. In particular, since the ubiquitination of TRAF proteins is required for their activation and proper TLR signaling, herpesviruses were shown to induce their deubiquitination. For instance, the HSV-1 US3 kinase inhibits TLR2 signaling pathway and the subsequent production of inflammatory cytokines by reducing TRAF6 ubiquitination, which is critical for its function in TLR2 signaling, and this requires the kinase activity of US3 (Sen et al., 2013). It is not clear how the US3-dependent kinase activity inhibits TRAF6 autoubiquitination. Understanding this molecular detail may reveal a mechanism mediating a crosstalk between phosphorylation and ubiquitination. The largest tegument protein of HSV-1, UL36, which is conserved among all herpesviruses, contains a N-terminal de-ubiquitinase motif known as UL36 ubiquitin-specific protease (UL36USP; Kattenhorn et al., 2005; Abaitua and O’Hare, 2008). This protein has been shown to be critical for HSV-1 replication as it deubiquitinates TRAF3, leading to the inhibition of TBK1 recruitment and ultimately a diminished IFN-β induction (Wang et al., 2013a). Furthermore, the HSV-1 ICP0 protein can trigger the translocation of the cellular E3 ubiquitin ligase ubiquitin specific peptidase 7 (USP7) from the nucleus to the cytoplasm, where it deubiquitinates TRAF6 to terminate the TLR-mediated NF-κB activation (Daubeuf et al., 2009). Also, EBV BPLF1 deubiquitinates TRAF6 to downregulate NF-κB signaling (Saito et al., 2013). These findings characterize viral proteins that counteract the ubiquitinating activity of TRAF molecules either *via* their intrinsic enzyme activity or *via* engaging cellular enzymes.

#### STING

STING is critical for the induction of the IFN response upon detection of viral DNA by intracellular DNA sensors. Therefore, herpesviruses acquired the ability to prevent STING-mediated antiviral responses. For instance, KSHV encodes four IRF homologs, namely vIRF1-4, which inhibit the host immune response by acting as transcriptional activators (for review, see Jacobs and Damania, 2011). KSHV vIRF1 was shown to interact with STING, blocking its interaction with TBK1. This negates STING phosphorylation and activation, and the subsequent induction of IFN-β, promoting KSHV replication (Ma et al., 2015). Similarly, HSV-1 ICP27 interacts with STING to block TBK1-mediated phosphorylation and activation of IRF3 (Christensen et al., 2016). A recent report showed that HSV-1 ICP34.5, a known neurovirulence factor, inhibits STING phosphorylation and activation likely *via* a targeted phosphatase activity, resulting in an increased viral replication (Pan et al., 2018). Given that multiple viral proteins from the same virus, e.g., HSV-1, target the cGAS-STING-IFN pathway for interference, it is a burning question how each individual viral protein contributes to the smoldered immune response and the elevated viral replication and pathogenesis thereof. Recombinant herpesvirus strains with exquisitely designed mutations will provide a powerful tool to answer this question.

Deubiquitination and subsequent inactivation of STING has also been shown to be triggered by herpesviruses to favorize their persistence. Sun et al. (2015), revealed that the absence of the deubiquitinase activity of the MHV68 ORF64 tegument protein is associated with an impaired delivery of the viral DNA into the nucleus. This ORF64 deficiency also triggers a STING-dependent antiviral signaling blocking the establishment of a latent infection *in vivo* (Sun et al., 2015). Interestingly, the gamma-herpesvirus ORF64 is a homolog of the alpha-herpesvirus UL36 protein that, in addition to its deubiquitinase activity, is also crucial for viral capsid maturation and subsequent tegumentation. A regulation of STING activation by HCMV has also been reported. Indeed, the HCMV-encoded UL48 deubiquitinase reduces the K63-linked ubiquitination of STING, thus blocking its activation and downstream antiviral signaling, while promoting HCMV-associated carcinogenesis (Kumari et al., 2017). These studies revealed that herpesviruses acquired the ability to inhibit STING activation to restrict the antiviral response upon viral DNA detection. However, in the case of HSV-1, a study reported an ambivalent link between STING and IFI16 and the virus-encoded proteins ICP0, ICP4, and US3, because these proteins appear to be required to stabilize IFI16 and STING in HSV-1-infected cells (Kalamvoki and Roizman, 2014). The observation that most herpesvirus-encoded deubiquitinating enzymes target STING for inactivation suggests a central role of STING in innate immune activation. Furthermore, the unique dynamic regulation of STING in trafficking from the ER membrane to the trans-Golgi network in innate immune signaling activation provides an opportunity to investigate the integration of organelles and PTMs in signal transduction.

### Post-translational Control of IRFs

IRF transcription factors are activated through phosphorylation by IKK-related kinases (TBK1 and IKKε), which induce their dimerization and subsequent translocation into the nucleus. Collaborating with other transcription factors of the NF-κB and AP-1 families, IRFs bind to interferon-sensitive response elements (ISRE) to activate the transcription of their target genes (e.g., IFN-β), establishing a potent antiviral response (for review, see Honda and Taniguchi, 2006). Given the pivotal role of the post-translational control of IRFs in the PRR-mediated antiviral signaling pathways, it is not surprising that herpesviruses subvert IRF activation or transcriptional activity by controlling their phosphorylation, ubiquitination, SUMOylation or acetylation.

In order to block the activation of IRFs by phosphorylation, herpesviruses adopted various strategies. For example, some herpesvirus strains encode kinases that are able to directly and aberrantly phosphorylate IRFs, which inhibits their transcriptional activity. The HSV-1-encoded kinase US3 interacts with and hyper-phosphorylates IRF3 at serine 175 and this abnormal phosphorylation blocks its activation (Wang et al., 2013b). Similarly, the EBV BGLF4 kinase phosphorylates IRF3 at Ser123, Ser173, and Thr180, in a region between the DNA binding and IRF association domains, suppressing the IRF3 signaling pathway (Wang et al., 2009). Varicella-Zoster Virus (VZV) ORF47 also aberrantly phosphorylates IRF3, preventing IRF3 phosphorylation at S396 and subsequent homodimerization (Vandevenne et al., 2011). Interestingly, this study also showed that the ORF47-mediated phosphorylation and inhibition of IRF3 dampens the IFN-β induction but not NF-κB activation, consistent with the selective inhibition on IRF3. Herpesviruses were also shown to deregulate cellular kinases responsible for the phosphorylation and activation of IRFs. The HSV-1 tegument protein US11 downregulates RLR signaling by interacting with RIG-I and MDA5, while impeding IRF3 activation by reducing its phosphorylation, dimerization and nuclear translocation (Xing et al., 2012). The HSV-1 protein ICP34.5 has been shown to directly interact with TBK1, blocking IRF3 phosphorylation immediately downstream of TBK1 and impeding the induction of type I IFNs to promote viral replication (Verpooten et al., 2009; Ma et al., 2012). Another study revealed that the MHV68-encoded ORF36 kinase selectively inhibits IRF3 by binding to its activated and phosphorylated form, facilitating the establishment of viral splenic latency *in vivo* (Hwang et al., 2009). Interestingly, the HSV-1-encoded serine protease VP24 downregulates the interferon stimulatory DNA-mediated phosphorylation and dimerization of IRF3 downstream of the cGAS-STING pathway, resulting in the inhibition of the IFN induction but not of the NF-κB activation (Zhang et al., 2016). KSHV also has adopted strategies to dampen the transcriptional activity of IRFs. For instance, the KSHV-encoded microRNA miR-K12-11 inhibits IRF3 phosphorylation by targeting and lowering the protein levels of the kinase IKKε (Liang et al., 2011). Noticeably, this study also showed that the absence of this microRNA enhanced KSHV reactivation induced by the infection with vesicular stomatitis virus. These studies demonstrated that viral proteins target IRF3 phosphorylation to block its activation and downstream signaling. Concerning IRF7, few studies revealed the control of IRF7 phosphorylation by herpesviruses. In particular, KSHV ORF45 competitively inhibits IRF7 phosphorylation by IKKε and TBK1, impairing IRF7 nuclear translocation and type I IFN induction (Liang et al., 2012; Zhu et al., 2002). These findings highlight the checkpoint role of IRF phosphorylation in regulating the antiviral IFN production.

In addition to inhibiting the phosphorylation-mediated activation of IRFs, herpesviruses also developed the ability to induce their ubiquitination and proteasomal degradation. The HSV-1 E3 ubiquitin ligase ICP0 promotes the ubiquitin/proteasome-mediated degradation of IRF3, thereby preventing IFN induction and subsequent antiviral immune defense (Melroe et al., 2004). Another study reported that VZV ORF61 directly interacts with activated IRF3 to promote the ubiquitination and downregulation of IRF3 (Zhu et al., 2011). Moreover, KSHV RTA possesses an intrinsic E3 ligase activity that ubiquitinates and induces the proteasomal degradation of IRF7 (Yu et al., 2005). The EBV latent membrane protein 1 (LMP1) was also shown to promote the TRAF6-mediated ubiquitination/proteasome-dependent degradation of IRF7 (Ning et al., 2008). The VZV protein ORF63 was shown to induce a reduction of IRF9 levels in a proteasome degradation-dependent manner (Verweij et al., 2015). These findings highlight the concept that phosphorylation and ubiquitination of IRFs are coupled by various viral and cellular proteins, forming a PTM-based signaling network.

The role of SUMOylation in the inhibition of IRFs activation by herpesviruses is not well understood yet. However, a study showed that EBV LMP1 induces IRF7 SUMOylation, reducing its turnover, increasing its nuclear retention, DNA binding and transcriptional activity (Bentz et al., 2012). Moreover, KSHV encodes a SUMO E3 ligase named basic region-leucine zipper (K-bZIP) that potentially targets the transcription factors IRF1, 2, and 7 (Chang et al., 2010; Chang et al., 2013). Other SUMO E3 ligases including EBV BRLF1, HSV-1 UL54/ICP27, and CMV UL69 were recently identified (De La Cruz-Herrera et al., 2018). These results emphasize that the SUMOylation of IRFs by herpesviruses can result in a loss of their transcriptional activity, suggesting that SUMOylation is a negative regulatory mechanism of IRF-mediated gene expression.

KSHV-encoded IRFs homologs have been shown to interfere in the assembly of the IFN-β enhanceosome. In support of this notion, vIRF1 directly interacts with p300 and reduces its histone acetyltransferase activity, leading to altered expression of cellular cytokines and consequently a diminished antiviral response (Li et al., 2000). vIRF1 can also block the interaction of cellular IRF3 with the enhanceosome, thus impeding the IFN induction. vIRF3 does not block IRF3 interaction with the IFN-β enhanceosome but interacts directly with it without impacting the acetylation of histones (Lubyova et al., 2004). These findings highlight the consensus of herpesvirus-mediated inhibition of IRF acetylation, to impair the assembly of a functional IFN-β enhanceosome and dampen the antiviral IFN response.

### Post-translational Control of NF-κB

The NF-κB family is composed of five transcription factors that underpin numerous innate immune signaling pathways and trigger the production of pro-inflammatory cytokines and chemokines (for review, see Zhang et al., 2017). The prototype NF-κB dimer is composed of two subunits, namely p50 and p65 (also known as RelA), which are often targeted by herpesviruses to control the inflammatory response. The HSV-1 kinase US3 is able to hyper-phosphorylate p65/RelA at serine 75, which blocks its nuclear translocation and consequently its transcriptional activity, leading to a reduced production of IL-8 (Wang K. et al., 2014). Another study showed that MHV68 hijacks RIG-I and MAVS to activate IKKβ that in turn phosphorylates RelA, thus facilitating the proteasome-mediated degradation of RelA and negating the antiviral cytokine production (Dong and Feng, 2011). Furthermore, HCMV UL26 blocks the phosphorylation and activation of IKKβ, which is the key step for IκB phosphorylation and NF-κB activation (Mathers et al., 2014).

NF-κB activation is a classic example in which kinase activity regulates the ubiquitination and subsequent degradation of a suppressor of a key transcription factor. Hence, upon reception of the upstream signal, the kinase IKKβ phosphorylates IκB, triggering IκB ubiquitination and its detachment from NF-κB which is now free to translocate into the nucleus and activate the transcription of its target genes (for review, see Chen and Chen, 2013). The activation mechanism of NF-κB constitutes a feed-forward mechanism involving these two modifications that is exploited by herpesviruses to reduce the inflammatory response. For instance, the E3 ligase of HSV-1 ICP0 was shown to dampen TNF-α-mediated NF-κB activation. To do that, ICP0 interacts directly with p50 and p65, blocks the nuclear translocation of p65, and induces the ubiquitination and proteasome-dependent degradation of p50, thus preventing the production of NF-κB-regulated pro-inflammatory cytokines (Zhang J. et al., 2013). Interestingly, ICP0 was also shown to trigger NF-κB activation by ubiquitinating IκBα during HSV-1 infection (Diao et al., 2005). The discrepancies between these studies are not self-evident, suggesting that the role of ICP0 may be contingent on the context of infection, e.g., temporal and viral parameters. Recently, Whitmer et al. (2015), reported that VZV ORF61 inhibits NF-κB activation by blocking IκB ubiquitination and degradation, thus retaining the p50–p65 dimer in the cytoplasm. Similarly, HSV-1 UL36USP deubiquitinates IκBα, preventing it from degradation and trapping p50–p65 in the cytoplasm (Ye et al., 2017). Another study showed that the MHV68-encoded ORF73, latency-associated nuclear antigen (LANA), interacts with ElonginC and Cullin5 *via* its suppressor of cytokine signaling (SOCS) motif to promote the ubiquitination and degradation of p65 (Rodrigues et al., 2009). The downregulation of NF-κB activation by MHV68 LANA is required for its latent infection. These studies expose cellular proteins that are specifically targeted by viral evasion proteins, reflecting their pivotal roles in NF-κB activation and inflammation.

### Post-translational Control of Cytokine- and IFN-Mediated Signaling

#### Janus Kinase (JAK)/Signal Transducer and Activator of Transcription (STAT)

Innate immune activation culminates in the production of type I IFNs and other inflammatory cytokines which, when bound to their cognate receptors, recruit and promote the activation of the non-receptor tyrosine kinase 2 (TYK2) and JAK1 kinases *via* proximity-induced autophosphorylation. Activated TYK2 and JAK1 kinase phosphorylate the transcription factors STAT1 and STAT2. Phosphorylated STAT molecules can undergo homo- or hetero-dimerization, in either case resulting in a functional nuclear localization signal that mediates their nuclear translocation. When STAT2 heterodimerizes with STAT1, the heterodimer binds to IRF9 and forms the ISGF3 complex, which transactivates the promoters containing ISREs (for review, see Villarino et al., 2017). Not surprisingly, herpesviruses deploy diverse strategies to interfere with the phosphorylation-mediated activation of the JAK/STAT pathway. HSV-1 infection induces the expression of SOCS1 and SOCS3 proteins to inhibit JAK phosphorylation, and this process requires the viral proteins VR3, UL13 and UL41 (Chee and Roizman, 2004; Yokota et al., 2004; Sato et al., 2017). Moreover, HSV-1 ICP27 downregulates STAT1 phosphorylation and subsequent accumulation in the nucleus in response to IFN-α (Johnson et al., 2008). VZV ORF63 has also been shown to interfere with JAK/STAT signaling by reducing the IFN-induced phosphorylation of STAT2 and mediating the degradation of IRF9 (Verweij et al., 2015). In human B cells, the EBV LMP1 directly interacts with TYK2, which blocks its phosphorylation and activation, thereby inhibiting the IFN response (Geiger and Martin, 2006). Another EBV protein, BZLF1, was shown to inhibit IFN-gamma-induced STAT1 tyrosine phosphorylation and nuclear translocation (Morrison et al., 2001). Finally, HCMV antagonizes STAT2 phosphorylation and activation (Le et al., 2008). Overall, these viral proteins primarily target the phosphorylation and nuclear translocation of STAT transcription factors to derail the IFN response.

The disruption of the JAK/STAT pathway by HCMV was reported 20 years ago when Miller et al. (1998), reported a virus-associated alteration of JAK levels. Subsequent studies revealed the ability of CMV to induce STAT ubiquitination and proteasome-dependent degradation. Indeed, MCMV pM27 was shown to induce STAT2 ubiquitination and degradation by the proteasome, likely through its interaction with the cellular protein damage specific DNA binding protein 1 (DDB1), known to be part of a ubiquitin-ligase complex (Trilling et al., 2011). In addition, this study showed that HCMV also induces the proteasome-mediated degradation of STAT2 but UL27, the HCMV-encoded homolog of MCMV pM27, is insufficient to downregulate STAT2 and is incapable of binding to DDB1. In MCMV-infected mice, pM27-mediated inhibition of STAT2 is essential for efficient MCMV replication, while the remaining activity of STAT2 is critical for the survival of the host (Zimmermann et al., 2005; Le-Trilling et al., 2018). These studies highlight the fact that herpesviruses acquired the ability to prevent further cytokine- and IFN-mediated viral clearance by manipulating the JAK/STAT pathway.

### Post-translational Control by ISGylation

PRR-mediated innate immune activation culminates in the production of type I IFNs that trigger the activation of a transcriptional program of so-called interferon-stimulated genes (ISGs). ISG15 is a ubiquitin-like 17 kDa protein that can be free in the cytoplasm or covalently conjugated to its target proteins in a reversible process termed ISGylation (Haas et al., 1987; Loeb and Haas, 1992; Narasimhan et al., 1996; Der et al., 1998; Potter et al., 1999; Hemelaar et al., 2004). ISGylation relies on the action of three enzymes catalyzing the conjugation and ligation of ISG15, named ISG15-activating enzyme (E1), ISG15-conjugating enzyme (E2), and ISG15-ligating enzyme (E3) (for review, see Durfee and Huibregtse, 2012). It has been established that ISGylation targets primarily, but not only newly translated proteins (Durfee et al., 2010). The exact consequences of ISGylation on the function of its target proteins remain elusive because it has been shown that ISGylation can restrict the ubiquitin system and downregulate protein degradation by the proteasomal pathway, thereby increasing protein stability (Okumura et al., 2008; Fan et al., 2015). However, ISGylation can also increase protein degradation by selective autophagy (Nakashima et al., 2015). In the context of antiviral innate immune signaling, it is now established that ISGylation plays an important role since ISGylation can target cellular or viral proteins involved in these pathways. Consistent with this, ISG15 and enzymes catalyzing ISGylation are upregulated by IFNs (Malakhov et al., 2003; Thaiss et al., 2016; Cai et al., 2017; Tecalco Cruz and Mejia-Barreto, 2017).

The role of ISGylation in antiviral signaling and immune evasion has been clearly established in the context of Influenza virus infection in particular. Hence, numerous studies revealed the importance of the ISGylation of viral proteins in antiviral signaling and the evasion mechanisms adopted by the virus to promote its replication. The non-structural protein 1 of Influenza B virus (NSB1) was identified as the first viral protein able to bind ISG15 (Yuan and Krug, 2001). Then, several virus-encoded proteins were shown to be able to bind ISG15 or remove it from target proteins (for review, see Zhao et al., 2013). Interestingly, NS1B was shown to restrict the ISGylation-mediated antiviral activity by sequestering ISGylated viral nucleoproteins which are responsible for the inhibition of viral protein synthesis and replication (Zhao C. et al., 2016).

ISG15 has been shown to target key components of innate immunity signaling pathways, e.g., RIG-I, STAT1, and IRF3, which promotes innate immune activation and thwart the replication of diverse viruses. Within the herpesvirus family, these include HSV-1, HCMV, and MHV68, prototypical herpesviruses of the α, β, and γ subfamilies, respectively (for review, see Morales and Lenschow, 2013). Specifically, free ISG15 was shown to promote the interaction between RIG-I and the autophagic protein p62, targeting RIG-I for degradation by selective autophagy (Du et al., 2018). In contrast, ISGylation stabilizes IRF3 by inhibiting its ubiquitination and degradation (Shi et al., 2010), and a similar effect of ISGylation on STAT1 was reported (Ganesan et al., 2016). These studies highlight distinct roles of ISG15 in early (RIG-I activation, IFN induction) and late (STAT1, IFN stimulation) stages of the IFN response, i.e., restricting early IFN induction and boosting IFN-stimulated effect. Although little is known about the mechanism by which herpesviruses exploit or evade the ISGylation-mediated immune defense system, a study carried out in ISG15-deficient mice showed that ISG15 has a protective effect against HSV-1 infection, suggesting its antiviral role against herpesviruses (Lenschow et al., 2007). Later, Jacobs et al. (2015) demonstrated that, upon TLR3 activation, KSHV vIRF1 interacts with the ISG15 E3 ligase, HECT and RLD domain-containing E3 ubiquitin ligase 5 (HERC5), leading to a global decrease in ISGylation of proteins in infected cells. Interestingly, vIRF1 is itself a target of ISGylation. The diminished level of cellular ISGylation found in this study was associated with reduced levels of IRF3, another known target of ISG15, supporting the conclusion that ISGylation stabilizes proteins (Jacobs et al., 2015). During HCMV infection, ISG15 inhibits HCMV growth by downregulating viral gene expression. Specifically, ISGylation of UL26 modifies its stability and suppresses its ability to dampen NF-κB activation. Conversely, HCMV IE1 and UL26 suppress the infection-induced ISGylation, to counteract the ISG15-mediated immune defense (Kim et al., 2016). HCMV UL50 interacts with and induces the proteasomal degradation of UBE1L, an E1-activating enzyme for ISGylation. Furthermore, RNF170, an endoplasmic reticulum-associated ubiquitin E3 ligase, interacts with UL50 and promotes UL50-mediated UBE1L degradation *via* ubiquitination (Lee et al., 2018). As such, knockdown of ISG15 increases HCMV productive infection in cultured cells (Bianco and Mohr, 2017). Collectively, these studies showed that ISGylation promotes the host innate immune response and that herpesviruses target various components to suppress ISGylation. Despite the difficulty of apprehending and studying ISGylation process since it occurs predominantly during the translation of proteins, the importance of ISGylation in the context of Influenza virus infection could provide further working hypotheses to get a better understanding of the role of this PTM in innate immune responses and in the pathogenesis of herpesvirus infection.

## Concluding Remarks

The regulation of protein function is chiefly achieved *via* PTMs and components of the innate immune response are of no exception. Retrospectively, the process of activation of the NF-κB pathway is powered by two key PTMs, phosphorylation and ubiquitination. While the networks of innate immune signaling pathways are established, new components are being discovered at an accelerating pace. Studies from microbial infection and herpesviruses in particular, offer great opportunities to understand the regulation of these components in addition to the roles of those pathways in host defense and microbial pathogenesis.

In the realm of innate immune defense, conventional approaches employ the established pathways and components to identify microbial modifiers. These approaches have yielded fruitful findings and advanced enormously our understanding of virus-host interactions. However, new cutting-edge technologies such as CRISPR screen and high throughput sequencing may be integrated to revolutionize how we discover new players in host immune defense using herpesvirus infection as a model system. This has the potential to unravel new components and to a less extent, new pathways in innate immune defense. Enzymes catalyzing PTMs possess diverse substrates, thus forging biological links between multiple processes that are otherwise unconnected. The discovery of cellular GATs in innate immune defense is an example that may provide an intrinsic link between metabolism and innate immunity, given that GATs catalyze the synthesis of key cellular metabolites such as nucleotides, amino acids and glycoproteins. On the other hand, acetylation of proteins such as histones is highly dependent on the cellular Acetyl-CoA pool, linking gene expression to the metabolic status as well. How the innate immune response connects with other fundamental biological processes *via* PTMs is in its infancy at best. To date, most of the studies on herpesvirus-mediated modulation of PTMs have focused on phosphorylation, ubiquitination, SUMOylation, deamidation, acetylation and ISGylation, leaving the modulation of other PTMs such as methylation, succinylation, carbonylation, glycation, glutamylation, hydroxylation, citrullination, nitration, palmitoylation and sulfation poorly understood or yet to be studied. The advancement of high-resolution mass spectrometric analyses should soon allow the sensitive detection of these PTMs and their regulation by herpesviruses.

## Author Contributions

JC and PF conceived and designed the study. JC, YR, QL, XL, JZ, and PF wrote the manuscript.

## Conflict of Interest

The authors declare that the research was conducted in the absence of any commercial or financial relationships that could be construed as a potential conflict of interest.
